# AMWEst, a new thermostable and detergent-tolerant esterase retrieved from the Albian aquifer

**DOI:** 10.1007/s00253-023-12844-2

**Published:** 2024-01-10

**Authors:** Moussa Adjeroud, Mouloud Kecha, Juan-José Escuder-Rodríguez, Manuel Becerra, María-Isabel González-Siso

**Affiliations:** 1 Laboratoire de Mycologie, Département de Biologie Appliquée, de Biotechnologie Et de L’Activité Microbienne (LaMyBAM), Faculté Des Sciences de La Nature Et de La Vie, Université Des Frères Mentouri Constantine 1, 25000 Constantine, Algeria; 2 University of A Coruña, Grupo EXPRELA, Facultade de Ciencias, Centro Interdisciplinar de Química e Bioloxía (CICA), A Coruña, Spain; 3 Laboratoire de Microbiologie Appliquée, Faculté Des Sciences de La Nature Et de La Vie, Département de Microbiologie, Université de Bejaia, Campus Targa Ouzemmour, 6000 Bejaia, Algeria

**Keywords:** Metagenomics, Esterase, *S. cerevisiae*, Next-generation sequencing

## Abstract

**Abstract:**

A fosmid library was constructed with the metagenomic DNA from the high-temperature sediment-rich water of the Albian aquifer (Algeria). Functional screening of this library was subsequently done looking for genes encoding lipolytic enzymes. We identified a novel gene named AMWEst (1209 base pairs) encoding a protein of 402 amino acids with a predicted molecular weight of 43.44 kDa and conferring esterase activity. AMWEst was successfully overexpressed in the yeast mesophilic host *Saccharomyces cerevisiae*, and the expression system used proved to be efficient and produced sufficient activity for its biochemical characterization. Multiple sequence alignment indicated that AMWEst contained a conserved pentapeptide motif (Gly120-His121-Ser122-Gln123-Gly124). The optimum pH and temperature of the recombinant esterase AMWEst were 8 and 80 °C, respectively. Additionally, AMWEst showed higher activity towards short carbon substrates and showed maximum activity for *p*-nitrophenyl hexanoate (C6). Notably, AMWEst has a remarkable thermostability, and the enzyme retains almost maximum activity at 70 °C after incubation for 1 h. Moreover, enzyme activity was enhanced by high concentrations of SDS and Triton X-100 detergents.

**Key points:**

*• A novel thermostable esterase has been retrieved through functional metagenomics*

*• The esterase is detergent-tolerant, which is attractive for some applications*

*• The esterase can be expressed in a yeast mesophilic host to enhance its yield*

## Introduction

In recent years, metagenomics has enabled enormous advances in the field of microbial ecology. The spectacular progress that sequencing technologies have achieved now makes it possible to sequence the entire DNA of a sample and subsequently access all the functions of an ecosystem. This approach allows the discovery of new enzymes and to access the enormous potential of genes in a given ecosystem (La Métagénomique—Développements et Futures Applications. (EAN13: 9782759222957) | Librairie Quae: Des Livres Au Coeur Des Sciences 2015). The majority of them will be new, but sequence-driven analysis will only identify genes and proteins similar to those with known functions (Culligan et al. 2014). Functional metagenomics, which is not dependent on the sequences present in databases, remain a powerful tool for the discovery of new families of genes as well as their encoded proteins (Culligan et al. 2014). Many studies of functional metagenomics consisting on the creation of a bank of recombinant clones, each containing DNA fragments of appropriate size (based on the desired format of the library ranging from short- to long-fragment libraries) cloned in a compatible vector, and screening for the genes of interest, have been successfully performed (Cowan et al. 2005). Soil represents an important reservoir of new biocatalysts, and much metagenomic work reports the discovery of several enzymes of industrial interest (Souza et al. 2018), such as lipases (Lim et al. 2020) and esterases (Chen et al. 2021). Extreme environments are characterized by unusual physicochemical conditions; these ecosystems represent an important reservoir of new extremophilic microorganisms (Iacono et al. 2020) which are specifically adapted to these stressful conditions, such as high temperatures (Kumar et al. 2020). These extremophiles have, besides the obvious ecological interest, the advantage of producing extremozymes which function under extreme conditions, an important aptitude in the field of biotechnology (Mirete et al. 2016).

Lipolytic enzymes are widespread in nature and are found in different microbial communities residing in all types of environmental niches (Martínez-Martínez et al. 2013). Of those, esterases of high temperature ecosystems have received considerable attention due to their thermostability and the many biotechnological applications these biocatalysts have (Zarafeta et al. 2016). However, the use of enzymes in industrial processes requires certain specific characteristics (Suharti et al. 2021). Industrial processes using high temperatures require thermostability, as well as overall tolerance to protein destabilizing agents, such as organic solvents, metal ions, surfactants and others (Zarafeta et al. 2016). The discovery of new thermophilic and hyperthermophilic esterases from the soil metagenome not only offers an opportunity in biotechnological applications, but also makes it possible to better understand the functioning of these complex ecosystems, and to enrich the catalogue of functions available with new sequences.

From metagenomic studies, the literature reports a wide variety of esterases, resulting from microbial communities of very diverse origins such as soil (Lu et al. 2019; Lim et al. 2020) and hot springs (Ranjan et al. 2018; Sharma et al. 2020).

In this study, we aimed to construct a metagenomic DNA library from a desertic soil concerning a region of the Algerian Sahara and screen it for the presence of lipase/esterase encoding genes. Sediment samples were taken around a borehole in the Albian aquifer, since the deep waters of the Albian aquifer are susceptible to colonization by extremophilic microorganisms. Soil is considered a complex and rich ecosystem for the discovery of important new enzymes (Kumar et al. 2015). Before this work, the construction of a metagenomic bank and the functional screening had never been attempted for samples of Algerian soils. In this study, we report the identification of a new hyperthermophilic esterase from a metagenomic library, its heterologous expression and biochemical characterisation. The heterologous host chosen to express the gene is the yeast *Saccharomyces cerevisiae*. Much work reports the success of heterologous production of hyperthermozymes in mesophilic hosts and applying a yeast expression system (Suleiman et al. 2020). Advantages of overexpression of thermozymes in *S. cerevisiae* include that it allows a high yield of enzyme production and that thermal denaturation purification techniques are easy to perform and will only affect the mesophilic enzymes of the host (Bruins et al. 2001). Another advantage is the facilitation of the secretion of foreign proteins in the extracellular environment (Aza et al. 2021). On the other hand, the production of recombinant proteins in bacterial systems has certain drawbacks related to protein folding, being as bacteria are unable to perform post-translational modifications (Deckers et al. 2020).

## Materials and methods

### Sampling site description

**Fig. 1:**
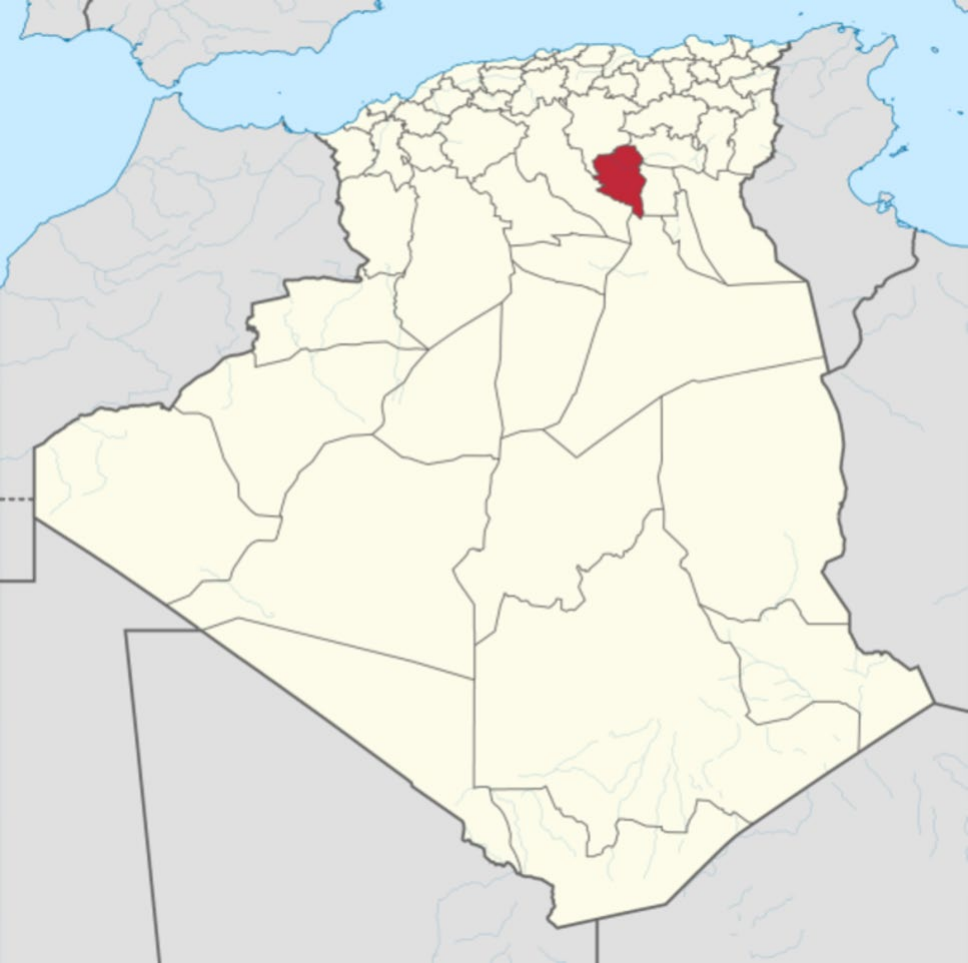
The localisation of the sampling site (red) in Ouled Djellal province in Algeria

The site chosen for the study concerns a region of the Algerian Sahara, precisely in the new Department of Ouled Djellal in south-eastern Algeria. The climate of the region is desertic. A new drilling of the Albian aquifer was carried out by a Chinese company in 2018 in the site named “El Wahass,” 7 km south of Ouled Djellal. The geographical location (34.44390°N, 5.13486°W) of the sampling site is shown in Fig. 1.

### Sampling and physicochemical analyses

**Fig. 2:**
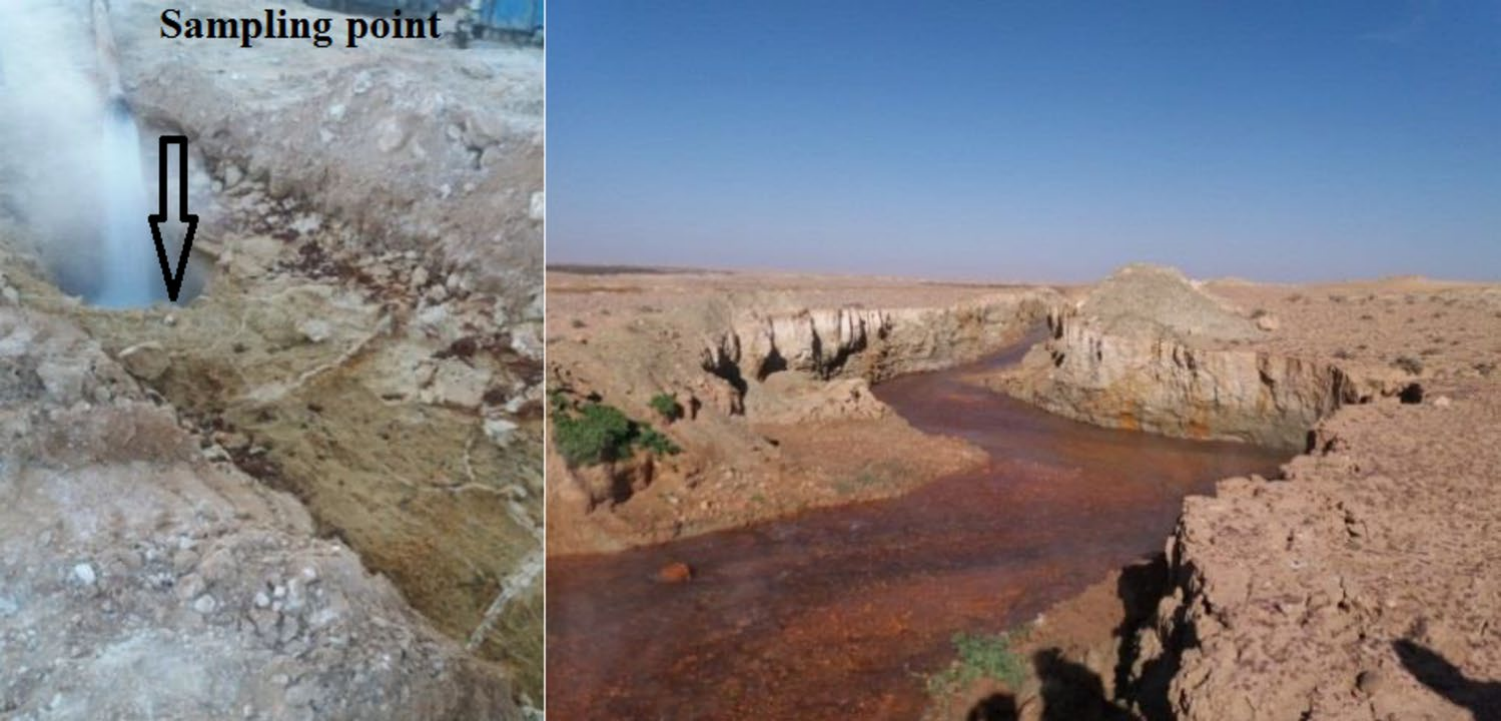
El Wahas sampling point in the Department of Ouled Djellal, Algeria

Sediment-rich water samples (10 cm deep) were taken around the borehole (85 °C, pH 7.18). These samples were taken in April 2019, with a sterilized polypropylene Falcon tube, in the middle of the small basin containing the waters of the Albian aquifer as shown in Fig. 2.

Physicochemical analyses of the water were performed in the Technical Institute for the Development of Saharian Agronomy, Biskra (Algeria), according to AFNOR (French Standardization Association) standards (https://www.afnor.org/en/. Accessed 8 Feb 2023).

### DNA isolation and construction of the metagenomic library

The extraction of nucleic acids was carried out following the protocol of the PowerMax soil DNA extraction kit (MO BIO Laboratories ref#12988–10; QiaGen, Netherlands). Total metagenomic DNA was extracted by the direct method, which consists of lysis of microbial cells within the soil matrix. To obtain a better yield, an additional concentration of the DNA was then carried out using a Millipore column of the Amicon Ultra 15-mL type. The purified high molecular–weight DNA was used to construct a metagenomic fosmid library with the pCC2FOS fosmid, using the Copy Control Fosmid Library Production kit (Epicentre, WI, USA), according to the manufacturer’s instructions. The library prepared comprised approximately 700 clones in the *Escherichia coli* strain EPI300-T1^R^. The metagenomic library clones were screened for lipolytic activity in LB-agar plates containing 1% tributyrin as substrate (López-López et al. 2015). The plates were incubated for 2 days at 37 °C and then further incubated at 60 °C for 48 h. The appearance of clear halos around the colonies indicates hydrolysis of tributyrin.

### Sequence pre-processing and assembly

The fosmid clones which showed lipolytic activity were grown overnight in liquid LB medium, supplemented with 12.5 μg/mL chloramphenicol and Copy Control Induction solution (Epicentre, WI, USA) to induce the clone to high-copy number. Fosmid DNA of each culture was extracted using FosmidMAX™ DNA Purification Kit (Epicentre, WI, USA). One microgram of the fosmid DNA was sequenced using Illumina PE150 in the Novogene company (London, UK). A total of 5,176,886 reads with a read size of 150 bp were generated. Reads with ambiguous bases (“Ns”), sequence duplicates and low-complexity sequences with score quality value > 25 were removed using PRINSEQ (Schmieder and Edwards 2011a). Removal of the readings corresponding to the pCC2FOS cloning vector (Genbank accession EU140752.1) and the genome of *Escherichia coli* (NC_000913) was undertaken using standalone Deconseq (version 0.4.3) with 90% coverage and 94% identity filtering options (Schmieder and Edwards 2011b). Remaining reads were then assembled using Spades software (Bankevich et al. 2012).

### Prediction and characterization of amino acid sequences

Open reading frames (ORFs) were predicted from contigs through the ORF finder program (https://www.ncbi.nlm.nih.gov/orffinder). ORFs were annotated by BLASTX using the NCBI protein (nr) database using an e-value 1e^−03^ and one best match was retained. Protein identities were performed using the BLASTP as well as PHI- and PSI-BLAST tools in the NCBI website (McGinnis and Madden 2004). A comparative BLAST analysis was performed on the Lipase Engineering Database (LED) platform (Fischer 2003). Molecular weights and isoelectric points (pI) were calculated using the Expasy molecular biology server (http://www.expasy.org/) (Gasteiger et al. 2005). Similarity searches of amino acid sequences were completed by protein BLAST (http://blast.ncbi.nlm.nih.gov). The amino acid sequence of the new identified lipolytic protein was aligned using COBALT multiple sequence alignment tool (www.ncbi.nlm.nih.gov/tools/cobalt/cobalt.cgi) with 12 top hits from BLASTP search, together depicted by ESPript 3.0 (http://espript.ibcp.fr/ESPript/cgi-bin/ESPript.cgi). Multiple alignments and phylogenetic tree were performed using the Muscle method with 35 lipolytic enzyme sequences from extended groups (I–XXXV) (Hitch and Clavel 2019), in MEGA tool version X (Kumar et al. 2018), with the Neighbor-Joining (NJ) method with 1000 bootstrap replicates; numbers at branching points indicate the percentage of consensus. The putative *N*-glycosylation sites were established with the NetNGlyc V1.0 server (http://www.cbs.dtu.dk/services/NetNGlyc/). The presence of signal peptide was detected with the bioinformatic tools Signal P (https://services.healthtech.dtu.dk/service.php?SignalP) and PrediSi (http://www.predisi.de/home.html) (Petersen et al. 2011).

### Subcloning

The gene encoding the new lipolytic enzyme was cloned in the YEpFLAG-1 plasmid (Eastman Kodak Company, Windsor, CO, USA) for heterologous expression in *S. cerevisiae* BJ3505 (*pep4::HIS3*, *prb-Δ1.6R HIS3*, *lys2-208*, *trp1-Δ101*, *ura 3–52*, *gal2*, *can1*) (Eastman Kodak Company, Windsor, CO, USA), a strain defective in protease PEP4, for better results in the expression of the cloned enzyme (Jones et al. 1982). To amplify the sequence of the gene, a primer pair consisting of LipMRecF (AAAGAAGAAGGGGTACCTTTGGATAAAAGAatgaaaattcttcggttcttc) and LipMRecR (TGGGACGCTCGACGGATCAGCGGCCGCTTActaatagcccttggcatagat) was used. The upper-case letters correspond to regions of homology to the cloning vector YEpFLAG-1 that allowed the cloning of the gene into the expression vector by homologous recombination. PCR cycling conditions were: initial denaturation (95 °C, 5 min); followed by 30 cycles of denaturation (95 °C, 1 min); annealing (65 °C, 1 min); extension (72 °C, 1 min); and a final cycle of 72 °C for 10 min. Phusion™ High Fidelity DNA polymerase (ThermoFisher Scientific, Waltham, MA, USA) was used. Cells of *S. cerevisiae* BJ3505 were co-transformed using the Frozen-EZ Yeast Transformation II Kit (Zymo Research, Irvine, CA, USA) with the PCR product and the YEpFLAG-1 plasmid, previously linearized by digestion with *Xho*I and *Sal*I (NZYTech, Lisbon, Portugal), and plated in a tryptophan-free complete medium (CM-trp) (Zitomer and Hall 1976) for selection of transformants containing the recombinant plasmid. Firstly, the clones were confirmed by determination of lipase activity in the extracellular medium as described below. Then, the recombinant plasmid was extracted from the yeast cells using the GeneJet Plasmid Miniprep Kit (ThermoFisher Scientific, Waltham, MA, USA) and propagated into the *E. coli* strain XL-1 Blue™ (Agilent Technologies, Santa Clara, CA, USA) to obtain enough recombinant DNA to verify the correctness of the construction by sequencing.

### Culture conditions

For expression and characterization of the recombinant protein, the recombinant strain was grown in Erlenmeyer flasks, filled up to 20% volume with YPHSM medium (8% bactopeptone, 1% yeast extract, 3% glycerol, and 1% dextrose, w/v) to improve stability of secreted recombinant proteins (López-López et al. 2015). Cultures were initiated by the addition of 1:20 volume of a 48-h pre-culture in CM-trp (Zitomer and Hall 1976) and grown for 4 days at 30 °C and 200 rpm. Supernatant was separated from cells by centrifugation, 13,000 rpm for 5 min, and concentrated using ultrafiltration with 10 kDa cut-off (Millipore).

### Lipase activity and biochemical characterization

Lipolytic activity was determined by a spectrophotometric method using *p*-nitrophenyl laurate as substrate (Fuciños et al. 2005). Briefly, 320 μL of activity buffer (50 mM Tris/HCl pH 8.5, 40 mM CaCl_2_) and 40 μL of *p*-nitrophenyl laurate stock solution (25 mM in ethanol) were incubated for 5 min at 60 °C. The addition of 40 μL of extracellular medium containing the recombinant enzyme initiated the reaction, which was stopped after 20 min of incubation at 60 °C with 100 μL of cold 1 M Na_2_CO_3_. The tubes were placed immediately on ice for 10 min, and then the precipitate was eliminated by centrifugation at 13,000 rpm for 10 min. A_400_ of the supernatant was measured. A blank was prepared using Milli-Q water instead of enzyme solution. The molar extinction coefficient of *p*-nitrophenol is 17,215 M^−1^ cm^−1^ under these conditions. One activity unit was defined as the amount of enzyme that produced 1 nmol of *p*-nitrophenol/min under standard assay conditions. The activities were expressed in EU/μL of culture medium.

The dependence of lipolytic activity on temperature was studied at different temperatures ranging from 40 to 90 °C. The dependence of lipolytic activity on pH was studied using different buffer systems at pH ranging from 4 to 9. In both cases, the reaction conditions are the same as those of the standard activity assay, and samples were performed in triplicate.

Thermostability was studied by measuring the residual activity after incubation of samples of ultrafiltrated extracellular medium containing recombinant enzyme at 70, 80 and 90 °C. Samples were taken at prefixed time points.

The specificity of the lipolytic enzyme against fatty acid esters of different chain length was measured using several substrates: *p*-nitrophenyl hexanoate (*p*NP6), *p*-nitrophenyl caprylate (*p*NP8), *p*-nitrophenyl laurate (*p*NP12) and *p*-nitrophenyl stearate (*p*NP18). The reaction conditions were the same as those used for the standard lipolytic activity measurement, but in this case, the substrate stock solution was prepared at a concentration of 5 mM in isopropanol to favour the dissolution of the substrates with longer fatty acid chains, and therefore more insoluble in water.

The stability against four surfactant agents was determined: SDS, CHAPS, Tween 20 and Triton X-100, at concentrations 0.1, 1 and 10% v/v for liquids, and 0.1, 1 and 10 mM for solids. The enzyme was incubated with the buffer and the surfactant agent for 1 h at 30 °C. As a control, the enzyme in buffer without surfactant agent was used. Samples were made in triplicate, and a blank was prepared for each case in which the enzyme was replaced by Milli-Q water.

The stability against four different commercial household detergents was determined: Dixan Aromatherapy, Marseille Soap (Eroski), Vanish Oxiaction Crystal White, and Somat 8 Actions. Solid detergents were prepared at a final concentration of 7 mg/mL, while liquid detergents were diluted 100 times, using the reaction buffer in both cases. Detergents may contain enzymes, for this reason, the tubes were previously placed in a bath at 80 °C for 30 min to inactivate detergent enzymes. The enzymatic extract of the lipase was then added and incubated for 1 h at 30 °C, to then measure the activity using the standard procedure. Enzyme incubated without detergents, and each detergent without enzyme, was used as controls. The samples were prepared in triplicate, as well as the control tubes with the enzyme.

The enzyme endoglycosidase H or Endo H (New England Biolabs, Ipswich, MA, USA) was used to carry out the deglycosylation reaction under denaturing conditions following the manufacturer’s recommended protocol. Five micrograms of protein was mixed with 1 µL of glycoprotein denaturing buffer 10 × , supplied with the enzyme, and Milli-Q water to complete 10 µL final volume, and then denatured at 100 °C for 10 min. Next, 2 µL of reaction buffer G5 10 × , 5 µL EndoH and Milli-Q water were added to reach a final volume of 20 µL. The reaction mixture was incubated at 37 °C for 1–2 h. SDS-PAGE was performed by the procedure described in Becerra et al. (1997). The marker NZY Colour Protein Marker II of molecular weights (NZYTech) was used as reference.

### Accession number

The gene sequence is available at the GenBank database under accession number ON513448 (AMWEst).

## Results

The results of the physicochemical analyses of the water samples from the Albien borehole at Ouled Djellal are summarized in Table 1. The waters of the Albian aquifer studied have a temperature of around 85 °C and a total mineralization of 2400 mg/L at pH 7.18. The physicochemical composition is very rich in sulphate and calcium and accompanied by a high conductivity of the water.

**Table 1:** The physico-chemical parameters of waters of the Albien, El Wahass, Ouled Djellal borehole

Physicochemical parameters	
Temperature (°C)	85
pH	7.18
Electrical conductivity (µS/cm)	3760
Total dissolved salts (mg/L)	2400
Chlorides (mg/L)	481.41
Sulphates (mg/L)	989.37
Calcium (mg/L)	312.6
Magnesium	-
Sodium (mg/L)	55.86
Potassium (mg/L)	33.23
Bicarbonates (mg/L)	292.87

### Construction and screening of the metagenomic library

**Fig. 3:**
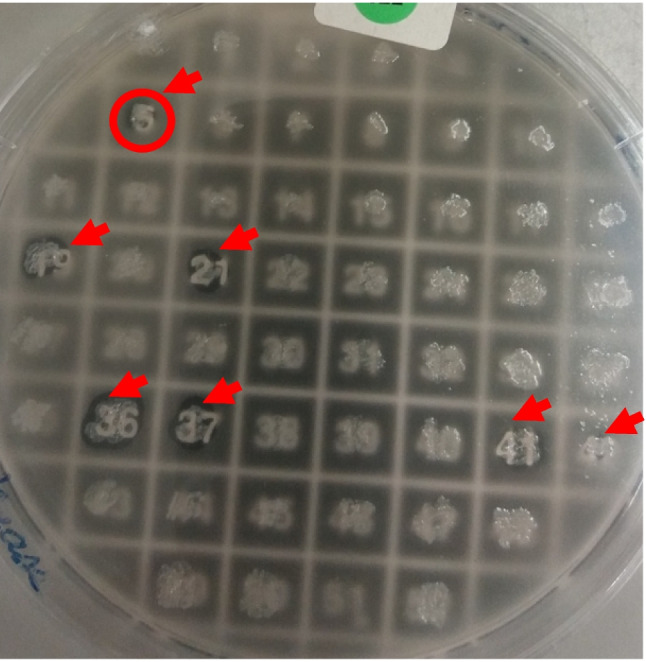
Screening plate containing the lipolytic positive clones after incubation for 24 h at 37 °C, and 48 h at 60 °C. Red arrows highlight the colonies where zones of hydrolysis of tributyrin were detected

The purified DNA isolated from the sediments showed the characteristics of a metagenomic DNA, and the size was around 35 kbp. The DNA concentration was 25 μg/mL; the nature of the sample (extreme desert soil, very high temperature water) made the extraction yields very low. A small library was successfully constructed, containing 700 clones, in the vector pCC2FOS. The clones were screened on tributyrin agar plates; we noted the presence of seven zones of hydrolysis of tributyrin around the colonies numbered 5, 19, 21, 36, 37, 41 and 42, as shown in Fig. 3. The positive clone number 5 was selected for further study. The DNA extraction yield of the fosmid containing the insert was very high, since the preculture contained an induction solution with arabinose.

### Sequence analysis of the cloned DNA

**Fig. 4:**
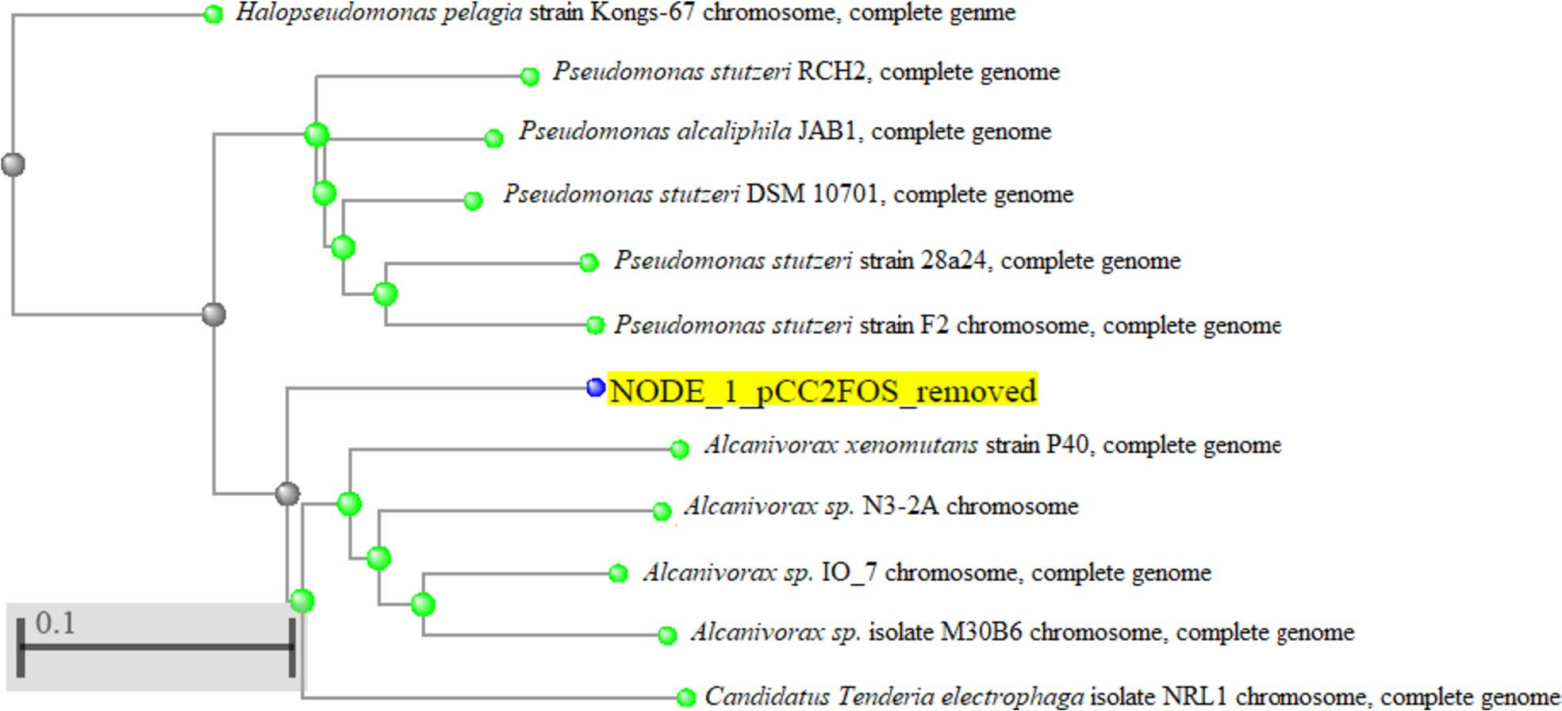
Phylogenetic tree of the insert containing the esterase gene by the “neighbor joining” method constructed in the NCBI portal

Sequencing reads (where each contig is called a “node”), containing the pCC2FOS sequence at both ends, were verified by performing a BLASTN and using the option to compare two sequences. Our insert contained precisely 35,000 base pairs. Comparison of the sequence with that of the nucleotide database using the BLASTN program proved homology to bacterial *Alcanivorax* group, with maximum homology of 76.09%. Figure 4 illustrates the phylogenetic tree of the BLASTN results after a pair alignment by the “neighbor joining” method built in the NCBI portal.

**Fig. 5:**
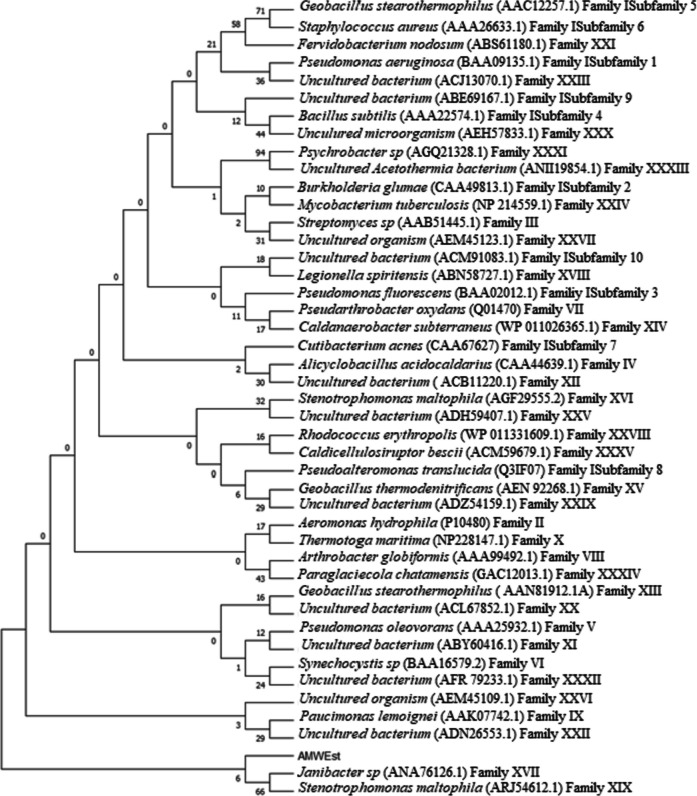
Phylogenetic tree based on amino acid sequence of AMWEst and 35 lipolytic enzyme sequences from extended groups (I–XXXV) (Hitch and Clavel 2019), in MEGA tool version X with the neighbor-joining (NJ) method. The numbers at the node indicate bootstrap percentages of 1000 replicates. Except for AMWEst, the protein sequences for previously identified families of bacterial lipolytic enzymes were retrieved from GenBank (http://www.ncbi.nlm.nih.gov. Accessed 8 Feb 2023)

ORF Finder software identified multiple genes inside the insert; a total of 271 open reading frames (ORFs) were predicted. Together with the NCBI platform BLASTP, it was possible to identify the different potential functions of the proteins encoded by these genes by comparing them with known proteins. The gene responsible of the lipolytic activity corresponded to ORF 224. This gene has 1209 base pairs and encodes a protein of 402 amino acids that was named AMWEst. The GC content of the gene is 58.89%. The predicted molecular mass of the protein is estimated at 43,441.28 Daltons and the isoelectric point (pI) at 4.71. Subsequent BLASTP analysis using the NCBI non-redundant protein database revealed that AMWEst showed moderate similarity (≤ 50%) to several lipolytic enzymes including the lactonizing lipase (GenBank: WP_015677081) from *Leptospira yanagawae* (identity 49%), the triacylglycerol lipase (GenBank: WP_020775775) from *Leptospira meyeri* (identity 49%), the lactonizing lipase (GenBank: EYF06121) from *Chondromyces apiculatus* DSM 436 (identity 49%), and the triacylglycerol esterase/lipase EstA (alpha/beta hydrolase family) (GenBank: MBB3048100) from *Litorivivens lipolytica* (identity 44%). This leads us to infer that AMWEst is a new enzyme from an unknown microorganism of prokaryotic origin that has not been cultured. Phylogenetic analysis using amino acids sequences of AMWEst and other lipases/esterases representing 35 different lipase/esterase families (I–XXXV) revealed that AMWEst belonged to the clade comprising the lipolytic family XVII and XIX enzymes (Fig. 5). The SignalP-5.0 program allowed the prediction of a peptide signal sequence whose cleavage site is located between position 22 and 23: LHA-EQ with a probability of 0.9223. The presence of a signal peptide leads us to conclude that the enzyme is directed to the secretion pathway (Álvarez-Cao et al. 2019). NetNGlyc V1.0 server allowed us to locate three sites of N-Glycosylation in the protein sequence.

**Fig. 6:**
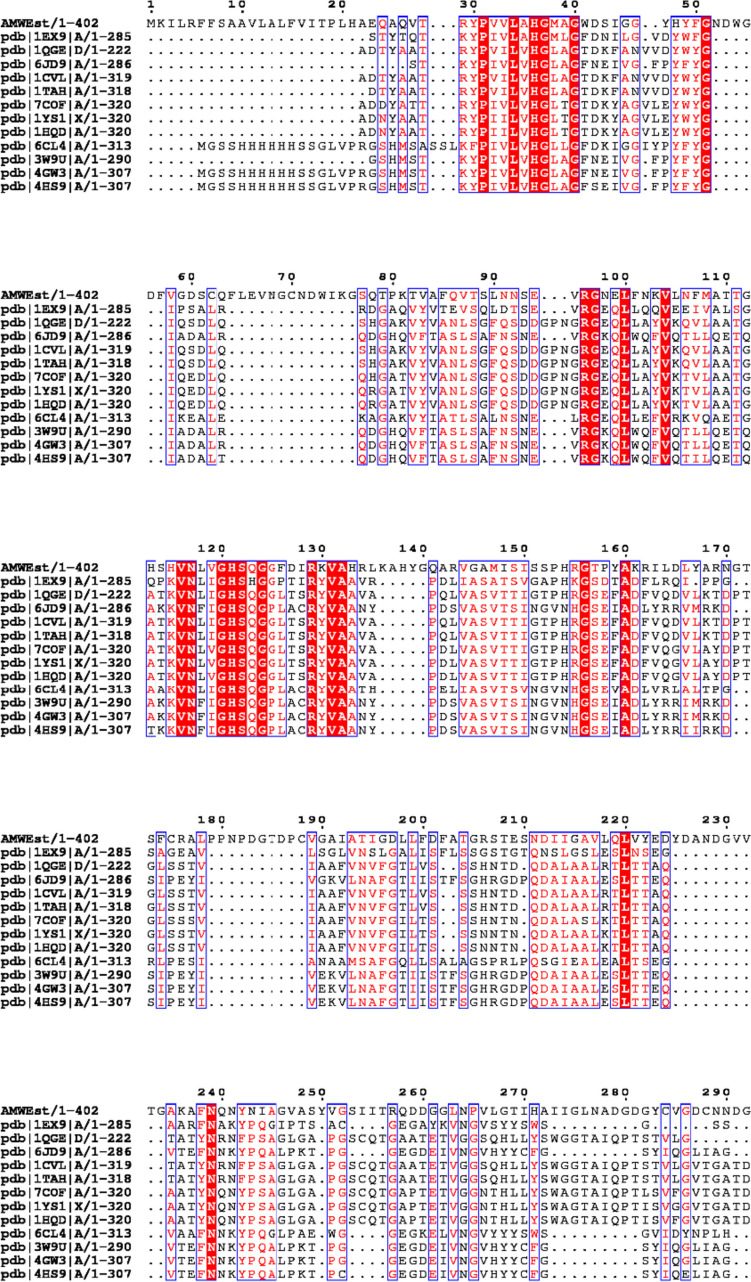
Multiple sequence alignment between AMWEst and other closely related lipolytic enzymes: 1EX9, crystal structure of the *Pseudomonas aeruginosa* lipase; 1QGE, new crystal form of *Pseudomonas glumae*; 6JD9, *Proteus mirabilis* lipase mutant; 1CVL, crystal structure of bacterial lipase from *Chromobacterium viscosum* ATCC 6918; 1TAH, crystal structure of triacylglycerol lipase from *Pseudomonas glumae*; 7COF, cholesterol esterase from *Burkholderia stabilis* (orthorhombic crystal form); 1YS1, *Burkholderia cepacia* lipase; 1HQD, *Pseudomonas cepacia* Lipase; 6CL4, Lipase from metagenomics (uncultured bacterium); 3W9U, Crystal structure of Lipk107 *Proteus mirabilis* HI4320; 4GW3, crystal structure of the lipase from *Proteus mirabilis*; 4HS9, methanol tolerant mutant of the *Proteus mirabilis* lipase

An alignment of multiple sequences (MSA) was performed with 12 sequences closely related (Fig. 6). The results showed that AMWEst contained a conserved pentapeptide motif (Gly120-His121-Ser122-Gln123-Gly124), which is a feature commonly found in esterases and typical of the α/β-hydrolase superfamily. From the MSA of AMWEst, numerous conserved regions were found such as PXXL (31–34), LXHGXXG (34–40), RG (96–98), KVN (115–117), RXVA (129–132), and GSEXA (156–160).

BLASTp analysis carried out against lipase engineering database (LED) with a cut-off e-value of 3e − 18, which revealed that the identified esterase AMWEst from El Wahass sediment metagenome is assigned to the superfamily “abH15” containing *Burkholderia* lipases with the homologous family *Burkholderia cepaciae* “abH15.02,” with a percentage identity of 33% to *S. cerevisiae* (abH15.03), lipase 2 like (23%), *Staphylococcus aureus* lipase like (abH15.01) (24%), *Streptomyces* lipases (abH16.01) (25%), *Bacillus* lipases (abH18.01) (33%), *Chloroflexus aurantiacus* lipase (abH17.01) (32%) and pancreatic lipases (abH20.03) (32%).

### Heterologous expression and biochemical characterization of AMWEst

Secretion of heterologous proteins in *S. cerevisiae* is affected by a variety of genetic and environmental factors such as culture conditions and others. Since the secretion of endogenous yeast proteins is low, the secretion of heterologous yeast proteins is particularly desirable (Wirajana et al. 2016). To overexpress AMWEst in *S. cerevisiae* BJ3505, the gene was amplified from the positive fosmid with gene-specific primers, and cloned into the yeast expression vector YEpFLAG-1, under the control of the *ADH2* promoter (Schuster et al. 2000). Plasmid YEpFLAG-1 is a yeast vector for the extracellular secretion of N-terminal FLAG fusion proteins in *S. cerevisiae*. YEpFLAG-1 also contains the *TRP1* gene for selection of yeast transformants (Yoshinaka and Kawai 2012). The successful expression and secretion of proteins of prokaryotic origin from this system is well documented (López-López et al. 2010, 2015). Lipase activity was measured from crude cell-free supernatants of the transformed yeast in YPHSM medium. As the AMWEst was readilly obtained in the extracellular medium without the need of further purification methods, the expression system used proved to be efficient and cost effective, producing sufficient activity for biochemical characterization. This confirmed that the ORF selected is the one responsible for the lipolytic activity shown by the fosmid clone. The lipolytic activity observed was relatively high, both for the extracellular and intracellular measurements, performed after 72 h of culture in YPHSM medium.

### Determination of substrate specificity and kinetic parameters

**Fig. 7:**
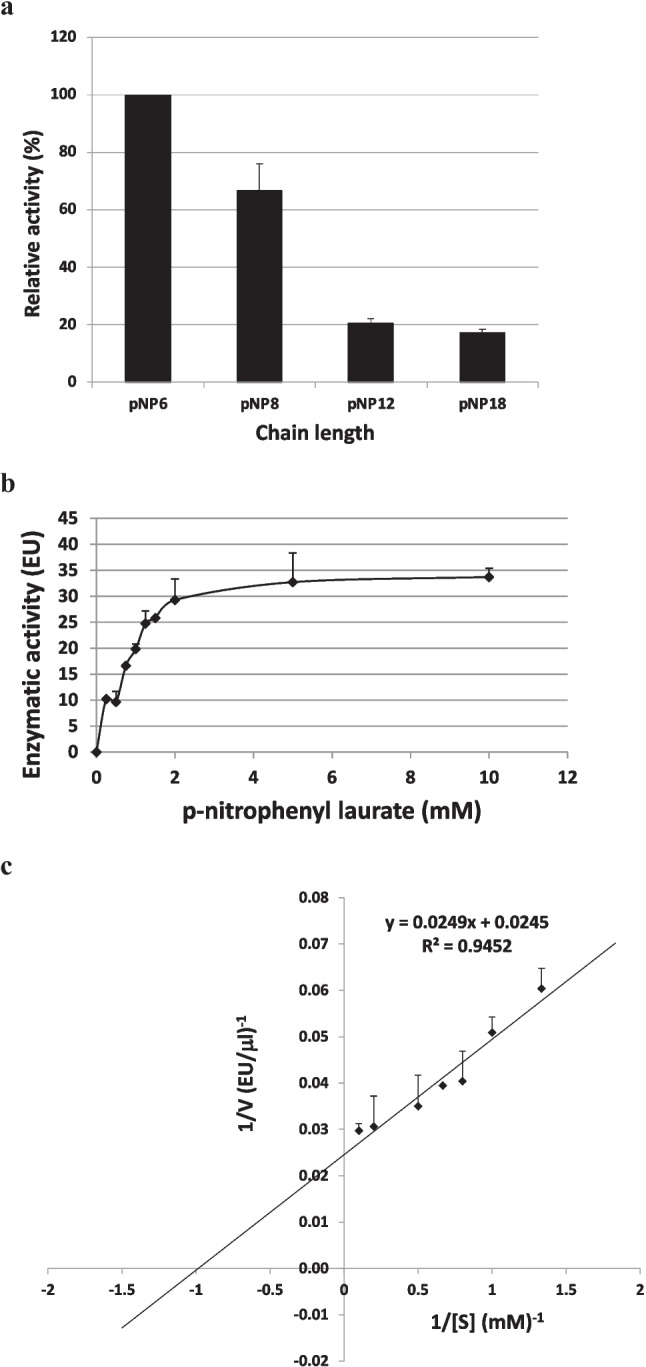
**a** Determination of the substrate specificity. 100% activity is 56.5 EU/μL. Data are the mean of three independent experiments. *p*NP6: *p*-nitrophenyl hexanoate; *p*NP8: *p*-nitrophenyl caprylate; *p*NP12: *p*-nitrophenyl laurate; *p*NP18: *p*-nitrophenyl stearate. **b** Michaelis–Menten kinetics for AMWEst using *p*-nitrophenyl laurate as substrate. Data are the mean of three independent experiments. **c** Lineweaver–Burk plot for AMWEst using *p*-nitrophenyl laurate as substrate. Data are the mean of three independent experiments. Standard activity assays were performed at 60 °C, pH 8.5 and 20 min incubation time

The determination of the substrate specificity was carried out under standard determination conditions of the enzymatic activity (Fuciños et al. 2005). The results are shown in Fig. 7a. The maximum activity, which was adjusted to the relative activity of 100%, was recorded for the short chain substrate *p*-nitrophenyl hexanoate (C6). The activity is relatively high for the *p*-nitrophenyl caprylate substrate (C8). We found that the activity with *p*-nitrophenyl hexanoate is more than four times higher than the activity with *p*-nitrophenyl laurate and *p*-nitrophenyl stearate, which confirms that the AMWEst enzyme is indeed an esterase, not a true lipase. To measure the variation of the reaction rate as a function of the substrate concentration, *p*-nitrophenyl laurate was used as substrate in a concentration range of 0.1–10 mM. The reaction conditions were the same as for the standard activity assay (Fuciños et al. 2005). The activity values for each of the concentrations were adjusted to a Michaelis–Menten kinetics (Fig. 7b). Kinetic parameters were 1.02 mM (*K*_*M*_) and 40.8 EU/µL (*V*_*max*_), and they were estimated on the Lineweaver–Burk plot (Fig. 7c).

### Biochemical properties of the esterase AMWEst

**Fig. 8:**
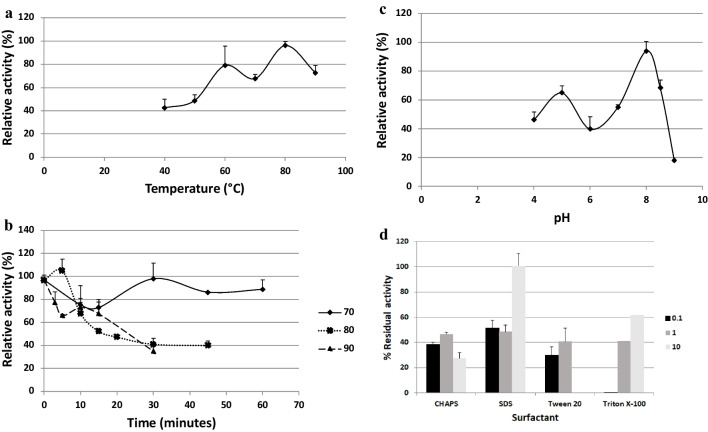
**a** Determination of the optimal temperature for AMWEst. 100% activity is 30.6 EU/μL. **b** Determination of the thermostability of AMWEst at 70, 80 and 90 °C. One hundred per cent activity is 17.6 EU/μL. **c** Determination of the optimal pH of AMWEst. One hundred per cent activity is 49.5 EU/μL. **d** Stability of AMWEst against the surfactants CHAPS, SDS, Tween 20 and Triton X-100 at different concentrations (0.1, 1 and 10% v/v for Tween 20 and Triton X-100 and 0.1, 1 and 10 mM for CHAPS and SDS). The percentages are referred to the control without surfactant that is the 100% activity (33.1 EU/μL). In all cases, data are the mean of three independent experiments. Standard activity assays were performed at 60 °C, pH 8.5 and 20-min incubation time, except the specific variations of temperature to find the optimal temperature and the specific variations of pH to find the optimal pH. All assays were performed using *p*-nitrophenyl laurate as the substrate

The effect of temperature and thermostability on lipolytic activity was determined using *p*-nitrophenyl laurate as a substrate at pH 8.5 in the temperature range 40–90 °C as shown in Fig. 8 a and b. The values obtained for each temperature were converted into relative activities. Figure 8 a shows a considerable increase in enzyme activity as a function of temperature. The maximum activity is measured at 80 °C. From 80 °C on, the activity gradually decreases. It is remarkable that the activity at 90 °C decreases by only 30%. At 70 °C, the enzyme is at 65% of its maximum activity.

The thermostability of AMWEst was assessed by determining the residual activity after incubation of the samples of the ultrafiltered extracellular medium containing the recombinant enzyme at 70, 80 and 90 °C, at predetermined time intervals. The results demonstrated a remarkable thermostability of the recombinant enzyme AMWEst (Fig. 8b). AMWEst retains almost maximum activity at 70 °C after incubation for 1 h. At 80 °C, activity is slightly above optimal after 5-min incubation. After 50-min incubation at 80 °C, AMWEst retains 40% of its activity. In the graph, we can see how the esterase activity decreases over time, the drop being much more drastic when exposed to very high temperatures, such as 90 °C. This is due to a denaturation of the exposed enzyme at high temperatures for prolonged periods. At 90 °C, we noticed a decrease in activity over time; AMWEst lost 70% of its activity after 30 min of incubation.

Figure 8 c shows that the optimal activity of AMWEst is located at pH between 7.5 and 8.5 where 80% of the activity is retained at the assay temperature (60 °C). At pH values above nine, the activity of the enzyme is low, and in addition, AMWEst did not retain good stability in the pH range (8.0 to 9.0); this indicated that it was a neutral stable enzyme. At pH 4.0, AMWEst retains 45% of its activity, and at pH 5.0, more than 60% of the activity is retained.

Regarding stability against surfactants, Fig. 8d shows that the presence of non-ionic surfactants such as Tween 20 or Triton X-100 caused inhibition of AMWEst activity, especially at low concentrations. Tween 20 (0.1%) caused 70% loss of enzymatic activity, and 0.1% Triton X-100 completely inactivated AMWEst. By increasing the concentration of Triton X-100, the enzymatic activity clearly increases, and at 10% concentration, the esterase AMWEst is 60% active. It is worth pointing out that the addition of 10% anionic sodium dodecyl sulfate (SDS) detergent has no significant effect on AMWEst activity as it retains 100% of its activity. On the other hand, the concentrations of 0.1 and 1%, negatively influenced the activity which decreased by 50%. The stability of AMWEst is remarkable, which was even boosted by high concentrations of SDS and Triton X-100. Regarding zwitterionic detergents, CHAPS caused AMWEst esterase activity inhibition especially at high concentrations.

**Fig. 9:**
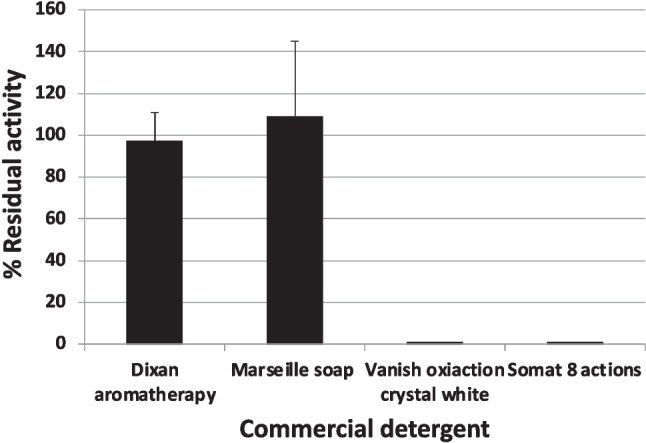
Stability of AMWEst against commercial detergents. The percentages are referred to the control without detergent that is the 100% (34.5 EU/μL). Data are the mean of three independent experiments

The stability of the enzyme AMWEst was also tested with four different commercial laundry and dishwasher detergents: Dixan Aromatherapy and Eroski Marseille soap (liquid), Vanish Oxiaction and Somat 8 actions (in powder). The results obtained are presented in Fig. 9. The results show that AMWEst is stable in liquid detergents; with Marseille soap, even the activity of the enzyme increases up to about 110%, while Dixan aromatherapy detergent is also compatible since the activity is mostly retained. In contrast, we found that the solid detergents Vanish Oxiaction and Somat 8 actions completely inhibit AMWEst activity.

**Fig. 10:**
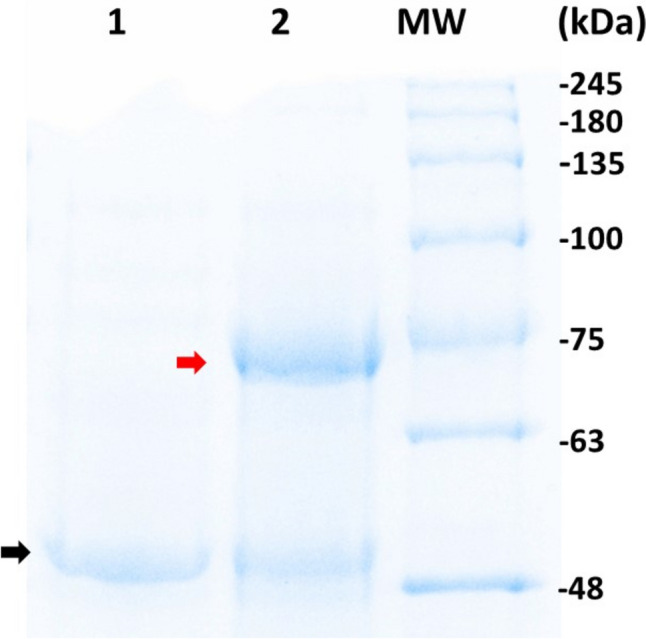
Analysis by SDS-PAGE. Five micrograms of protein was loaded before (1) and after (2) EndoH treatment. MW, NZYcolour Protein Marker II (NZYTech). Black arrow, glycosylated AMWEst. Red arrow, product of aggregation of two deglycosylated AMWEst molecules

As we detected three potential *N*-glycosylation sites in the sequence analysis, the putative glycosilation of AMWest was tested by treatment with Endoglycosidase H. A band of about 50 kDa is observed in the SDS-PAGE gel (Fig. 10, black arrow), which is slightly larger than the estimated molecular weight for AMWEst (43.4 kDa). Since it has three putative N-glycosylation sites, this size difference may be due to the glycosylation suffered during secretion in yeast. Interestingly, after EndoH treatment, a new band of about 75 kDa appears (Fig. 10, red arrow) that could be the result of the aggregation of two deglycosylated AMWEst esterase molecules.

## Discussion

In the current work, we constructed a metagenomic library using DNA isolated from a thermal environmental sample from desert soil in the Algerian Sahara. The methodology implemented to extract the metagenomic DNA consisted of in situ cell lysis. Hard cell lysis uses chemical and mechanical methods where cells are broken by shaking with glass beads. It has been reported that the direct method employed in our protocol gives better yield compared to the indirect methods (Lakay et al. 2007). Our attempts to isolate high quality genomic DNA have encountered some difficulties associated with low yield and poor-quality DNA from desert sediments, which is directly related to the nature of the sample, exposed to environmental stress imposed by solar radiation and other factors, such as desiccation, temperature or limited availability of nutrients, contributing to low microbial community abundance (Tanner et al. 2020), which had repercussions on the size of our library that consisted of only 700 clones with an average insert size of 35 kb. Despite its small size, after functional screening of the metagenomic library via hydrolysis of tributyrin on LB medium, the culture independent approach allowed us the identification and characterization of a new thermostable esterase, AMWEst. In a previous work of functional metagenomics, lipase screening from the Lobios thermal spring in Galicia (Spain), the isolation of six positive clones out of a total of 11,600 clones is reported (López-López et al. 2015), and one positive clone was isolated by analyzing 21,000 clones in the Turban basin (Fan et al. 2012). A recent work shows that the incidence of new positive clones (lipase/esterase) is 1 hit per 17,325 clones (Ferrer et al. 2016). Despite this low rate of positive hits detection, we were able to identify up to seven positive lipase/esterase hits with a 700-clone sized library. Many parameters can compromise the functional metagenomic approach, such as the selection of the expression host. Although strains of *E. coli* are commonly used, some genes from environmental samples may not be expressed efficiently due to differences in the genetic code and codon usage bias, protein folding elements, post-translational modifications or active enzyme toxicity (Uchiyama and Miyazaki 2009). It has been reported that only 40% of thermophilic lipolytic enzymes present in a metagenomic library are recovered by functional screening when *E. coli* is used as a host (Gabor et al. 2004).

The AMWEst enzyme is indeed a novel biocatalyst not described elsewhere. The amino acid sequence derived for the AMWEst showed resemblance to serine hydrolases, their activities being based on a catalytic triad, comprising Ser-Asp/Glu-His with a consensus sequence (Gly-x-Ser-x-Gly) (Adetunji and Olaniran 2021). AMWEst contains the GxSxG motif in the form of GHSQG. This same motif is found in an esterase belonging to the family XXII in the new lipase classification update undertaken by Hitch and collaborators (Hitch and Clavel 2019), and EstD2 comes from a metagenomic library produced from the rhizosphere microbiome of multiple plants (Hitch and Clavel 2019). Another enzyme where the consensus sequence GHSQG is reported is PaLip, a representative of family I (Rojo 2019). In the phylogenetic tree, AMWEst together with the esterase/lipase uncultured bacterium and *Pseudomonas oleovorans* forms a distinct subgroup in family I, V and family XXV. The prediction of AMWEst in the Lipase Engineering Database shows that the protein sequence is a new lipolytic enzyme belonging to the superfamily “abH15” containing the lipases of *Burkholderia* with the homologous family *Burkholderia cepaciae* “abH15.02”. The nomenclature abHn.m where abH stands for alpha beta hydrolase, followed by the number assigned to the superfamily *n*, separated from the number of the family of homologs *m* by a period. The identity percentage is 33%. The abH15.02 family includes 242 sequences including three proteins of the bacterial genus *Alcanivorax* and whose NCBI accession numbers are respectively 110834836, 196194968 and 196193133. Members of the genus *Alcanivorax* belong to a group of hydrocarbonoclastic bacteria known for their use of alkanes and other related compounds as a preferred carbon source. The genomic characteristics of the strain *Alcanivorax* sp. 24 isolated from marine plastic debris show that its 4,765,873 bp genome, containing 4239 coding sequences, revealed the presence of all the genomic characteristics involved in the degradation of alkanes (namely, two cytochrome P450, three alkane monooxygenases AlkB and two enzymes involved in the degradation of long chain AlmA alkanes) as well as other enzymes that may play a role in the biodegradation of other polymers such as polyhydroxybutyrate (Zadjelovic et al. 2020). Recent work reports the isolation of a new *Alcanivorax* sp. strain VBW004 from a shallow thermal vent in Azores, Portugal; the strain is very resistant to copper and can be used in the bioremediation of polluted soils (Ramasamy et al. 2020).

This study shows that the cloning and heterologous expression of the *AMWEst* gene in the mesophilic yeast *Sacharomyces cerevisiae* was very efficient and reflected the adaptation of the expression host to obtaining a high level of production of the recombinant protein. In the literature and from previous work, protein production based on heterologous genes may be low, especially if the genes expressed are from thermophilic bacteria due to the difference in the cytoplasmic environment and the high GC content of thermophilic genomes (Krefft et al. 2017). The chosen expression system did not negatively affect the expression levels of the recombinant gene; thus, the use of *S. cerevisiae* as a eukaryotic cell factory is appropriate in this scenario. Unlike various prokaryotic expression systems, *S. cerevisiae* possesses the ability to effect post-translational modifications and secretion, which greatly facilitates the purification steps. The advantages of overexpression of thermozymes in *S. cerevisiae* are that this yeast allows a high yield of extracellular enzyme production, and that thermal denaturation purification techniques are easy to perform since heat treatment only affects the mesophilic enzymes of the host (Bruins et al. 2001). Moreover, the production of recombinant proteins in *S. cerevisiae* can be improved by using new genetic tools and advanced cell engineering strategies (Liu et al. 2012; Aza et al. 2021). These advantages are in addition to the ability of *S. cerevisiae* to resist low pH and high osmotic pressure. Other studies also show the involvement of the alpha factor which is very efficient as signal for secretion of proteins (Liu et al. 2012). What is interesting from the present work is that the mesophilic yeast *S. cerevisiae*, widely used as a eukaryotic model suitable for the production of several industrial products and secondary metabolites (López-López et al. 2010; Schmoll and Dattenböck 2016), proved to be useful also for the production of thermozymes from genes of uncultured thermophilic organisms. Many studies report an advantageous expression in *S. cerevisiae* of genes from hyperthermophilic organisms compared to other expression systems, for example, recombinant ornithine carbamoyltransferase from *Pyrococcus furiosus* was as stable as the native enzyme when expressed in *S. cerevisiae* (Vieille and Zeikus 2001). In other previous works, a high potential of *S. cerevisiae* for secretion of the putative *Thermus thermophilus* HB27 YP_004875.1 esterase has been reported (López-López et al. 2010), as well as EstA from *Burkholderia gladioli* (Breinig et al. 2006)*.*

AMWEst showed preference for *p*-nitrophenyl hexanoate, while activity on longer chain substrates was lower. The recombinant enzyme was very effective against short-chain *p*-NP esters compared to other esterases reported in previous works, and with kinetic data relatively close to other thermostable esterases. Thus, Castilla and collaborators reported the value of the Michaelis–Menten Km constant of a new family of *Janibacter* sp. R02 esterase which is 0.873 mM (Castilla et al. 2017). For *Streptomyces lividans* TK24 esterase, EstA, Chang et al. reported *K*_*M*_ and *V*_*max*_ values of 0.34 mM and 16.4 ± 0.5 μM min^−1^ respectively, when the substrate was *p*NP2 (Chang et al. 2021). For an extremely thermostable esterase of *Pyrococcus furiosus*, Pf_Est, Mandelli et al. reported *K*_*M*_ and *V*_*max*_ values of 0.53 mmol/L and 6.5 × 10^−3^ U respectively, when the substrate was *p*-nitrophenyl palmitate (*p*NPP) (Mandelli et al. 2016). Compared to esterases from functional metagenomic studies, for LOB4Est, MLC3 and SLC5 esterases, López-López et al. and Ranjan et al., respectively, reported *K*_*M*_ and *V*_*max*_ values of 0.298 mM and 263.778 U/L, 134 μM and 14.55 μmol/min/mg and 196.5 μM and 2.393 μmol/min/mg when the substrate was firstly *p*-nitrophenyl laurate and *p*-nitrophenyl butyrate (López-López et al. 2015; Ranjan et al. 2018). Regarding the esterases in particular, functional at temperatures around 80 °C, as is the case for our study, the kinetic parameters of Est1 are 3.0 M for *K*_*M*_ and 31.2 U/mg for *V*_*max*_ (Lu et al. 2019). *K*_*M*_ and *V*_*max*_ are the two important parameters representative of the Michaelis–Menten kinetics. Most enzymes used in industrial processes show *K*_*M*_ values located in the range of 10^−1^ to 10^−5^ M (Barzkar et al. 2021).

The results of the study of the effect of temperature on AMWEst esterase activity show maximum activity measured at 80 °C. The same result (not shown) was obtained with the enzyme produced by the fosmid positive clone. That is, even when very low enzyme activity was detected in the recombinant *E. coli* clone, the method used allowed us to have information on the optimum temperature for the activity of the enzyme. The optimum activity temperature is close to the temperature of the ecosystem studied (85 °C). An interesting fact is that the optimum temperature of the recombinant enzyme is the same from the *E. coli* fosmid system and from the yeast expression system, and therefore, the use of a mesophilic heterologous expression system did not adversely affect the optimum esterase activity. Unlike other previous work which reports a decrease in the optimum temperature of the recombinant enzyme after expression in mesophilic hosts such as for example *Thermus* esterase where the lowest optimum temperature (40 °C) was found when *S. cerevisiae* was the host, while the native enzyme of *T. thermophilus* exhibits an optimum temperature at 80 °C (López-López et al. 2010). This suggests that AMWEst would be a better candidate for industrial processes operating at high temperature. These results are in addition to a lot of work on the characterization of esterase enzymes from hyperthermophilic microorganisms such as *T. thermophilus* HB27, whose optimum temperature is 80 °C (Fuciños et al. 2005). Other microorganisms, such as *Archaeoglobus fulgidus*, secrete esterases with an optimum temperature for activity of 80 °C (Manco et al. 2000). It has recently been shown that bacteria such as *Janibacter* sp. R02 secrete esterases with maximum activity at 80 °C (Castilla et al. 2017). *Pyrococcus furiosus* is one of the extremophiles producing highly thermostable esterases with an activity optimum of 80 °C (Mandelli et al. 2016). Our results add to the great deal of metagenomic work relating to the isolation of new genes encoding new biocatalysts (esterases) from sediment sources of thermal waters: Lobios hot springs in Spain (Miguel-Ruano et al. 2021); Azores Islands in Portugal and Göttingen in Germany (Leis et al. 2015) and Khir Ganga in India (Ranjan et al. 2018).

Regarding the thermal stability of extracellular enzymes produced by microorganisms, it has also been reported that hyperthermophilic proteins exhibit significantly reduced hydrophobic accessible surfaces compared to mesophilic proteins with the predominance of one or more proline residues located in the loop surface which reduce the thermal flexibility of the loop, and the enzyme becomes thermostable (Akassou 2018). This rigidity contributes to the protection against unfolding and preserves their catalytically active structure (Bruins et al. 2001). AMWEst has shown remarkable thermal stability at 90 and 80 °C with a half-life of more than 20 min at 90 °C and 15 min at 80 °C, respectively. The thermostability of AMWEst is greater than that reported for two thermostable esterases derived from a compost metagenome: Est1 and Est2 and which have a maximum activity at 80 and 70 °C. Est1 keeps only 50% of its activity after 15 min of incubation at 70 °C, Est2 keeps 35% of its activity at 80 °C after 30 min of incubation (Lu et al. 2019). However, our enzyme preserves 40% of its activity after 30 min of incubation at 80 °C and more than 35% at 90 °C. The enzyme was almost 100% stable at 70 °C, even after 60 min. The secondary structure of thermophilic proteins is rich in α helix and β sheet in comparison with mesophilic proteins, which considerably protect their structure against different denaturing conditions (Egamberdieva et al. 2021). Thirty-six per cent of the structure of AMWEst is composed of α helix and 11% of β sheet according to secondary structure predictions (data not shown) which confirms the data cited above.

Testing the stability of esterases and lipases to surfactants and detergents is important, since these biocatalysts are widely used in the formulation of these industrial products as an emulsifying agent at concentrations of 10 to 20% (Prazeres et al. 2006). It is well known that a diverse set of surfactants and solvents affect the enzymatic activities of esterases and lipases from various sources, although the magnitude is quite different. Even in the presence of 10% of Triton X-100 or SDS, AMWEst kept its stability. Our results converge with much work on the characterization of esterase enzymes from thermophilic and hyperthermophilic microorganisms which can be cultivated such as *Ureibacillus thermosphaericus* (Samoylova et al. 2018), *Geobacillus* sp. HBB-4 (Metin et al. 2006), *Bacillus licheniformis* (Bhardwaj et al. 2020), *Thermotoga maritima* (Levisson et al. 2007), *Geobacillus thermodenitrifcans* NG80-2 (Curci et al. 2019), *Bacillus pumilus* (Sharma et al. 2016) and *Bacillus aryabhattai* B8W22 (Zhang et al. 2019) especially for detergents, Triton X-100, CHAPS, Tween 20 and even from the construction of metagenomic libraries (Lewin et al. 2016; Pereira et al. 2017; Jayanath et al. 2018; Li et al. 2019). In many of the studies cited above, SDS detergent greatly reduces the enzymatic activity even at low concentrations. The stability of enzymes towards SDS is rarely reported in research papers. The work of Cherif et al. shows the tolerance of a lipolytic enzyme to SDS surfactant, at 1% concentrations (Cherif et al. 2011). In another study, Akmoussi-Toumi et al. successfully purified a lipase from a halophilic archaebacterium resistant to 1% SDS detergent (Akmoussi-Toumi et al. 2018). Ghati and Paul, in their work on the purification of an esterase from *Geobacillus* sp., demonstrated the tolerance of the enzyme to 0.5% of SDS (Ghati and Paul 2015). To put the data of stability of AMWEst with commercial detergents obtained in perspective, in similar experiments as developed by Cherif et al. and Akmoussi-Toumi et al., in which they tested the compatibility of its enzymes with various commercial detergents among which there were detergents of the Dixan brand, their enzymes maintained 80% of their activity (Cherif et al. 2011; Akmoussi-Toumi et al. 2018). In the same context, the work of Romdhane et al. on the fungal lipase of *Talaromyces thermophilus* reveals compatibility with Dixan detergent, and the enzyme retains 80% of its activity (Romdhane et al. 2010).

## Data Availability

The gene sequence is available at the GenBank database under accession number ON513448 (AMWEst).
